# Slippery Epidural ECoG Electrode for High-Performance Neural Recording and Interface

**DOI:** 10.3390/bios12111044

**Published:** 2022-11-18

**Authors:** Md Eshrat E. Alahi, Yonghong Liu, Sara Khademi, Anindya Nag, Hao Wang, Tianzhun Wu, Subhas Chandra Mukhopadhyay

**Affiliations:** 1 The Institute of Biomedical and Health Engineering, Shenzhen Institute of Advanced Technology, Chinese Academy of Sciences, Shenzhen 518055, China; 2 Institute of Polymeric Materials and Faculty of Polymer Engineering, Sahand University of Technology, Tabriz P.O. Box 51335/1996, Iran; 3 Faculty of Electrical and Computer Engineering, Technische Universität Dresden, 01062 Dresden, Germany; 4 Centre for Tactile Internet with Human-in-the-Loop (CeTI), Technische Universität Dresden, 01069 Dresden, Germany; 5 The School of Engineering, Macquarie University, Balaclava Rd, Macquarie Park, NSW 2109, Australia

**Keywords:** ECoG electrode, slippery coating, surface modification, Pt-gray, nanocone, neural interface

## Abstract

Chronic implantation of an epidural Electrocorticography (ECoG) electrode produces thickening of the dura mater and proliferation of the fibrosis around the interface sites, which is a significant concern for chronic neural ECoG recording applications used to monitor various neurodegenerative diseases. This study describes a new approach to developing a slippery liquid-infused porous surface (SLIPS) on the flexible ECoG electrode for a chronic neural interface with the advantage of increased cell adhesion. In the demonstration, the electrode was fabricated on the polyimide (PI) substrate, and platinum (Pt)-gray was used for creating the porous nanocone structure for infusing the silicone oil. The combination of nanocone and the infused slippery oil layer created the SLIPS coating, which has a low impedance (4.68 kΩ) level favourable for neural recording applications. The electrochemical impedance spectroscopy and equivalent circuit modelling also showed the effect of the coating on the recording site. The cytotoxicity study demonstrated that the coating does not have any cytotoxic potentiality; hence, it is biocompatible for human implantation. The in vivo (acute recording) neural recording on the rat model also confirmed that the noise level could be reduced significantly (nearly 50%) and is helpful for chronic ECoG recording for more extended neural signal recording applications.

## 1. Introduction

Neurological disorders or injuries of the central nervous system disturb any individual’s daily life [1]. The brain–computer interface (BCI) is the necessary tool to restore the disability problems of any individual [2]. For example, chronically implanted devices can be used to record the brain signal, which can be used for decoding the synthesized voice [3], controlling the movement of the computer cursor [4] and robotic prosthetic limbs [5]. Therefore, it is essential to combine the interface between the neural tissues and implantable devices for recording the neural signals seamlessly over a long period. Recently, many implantable electrodes with different shapes [6], materials, and integration techniques have been developed to integrate with the brain interface for recording the neural signals [7]. Neural signals can be recorded in three different ways, such as by Electroencephalography (EEG), Electrocorticography (ECoG), and intracortical electrode (IE), to communicate for BCI applications. A minimally invasive ECoG electrode can directly record the local field potential (LFP) from the cerebral cortex with direct contact with the brain. The ECoG electrode can be implanted directly on the dura mater by either subdural or epidural approaches. The epidural approach is better than the subdural approach due to the lower disruption of the brain tissues [8,9]. However, a scar can be seen around the electrode, which arises from acute insertion trauma (ACT) and chronic inflammatory body reactions. ACT can cause acute neural loss, which accelerates the formation of the glial sheath around the electrode [10,11]. Therefore, avoiding glial scar accumulation around the electrode recording sites and the substrate is crucial. 

When an electrode is implanted inside the brain to record the neural signal, the brain’s blood–brain barrier (BBB) is disrupted, showing a series of inflammatory responses for keeping the natural homeostasis of the brain upon the electrodes’ implantation. The inflammatory reactions trigger the degradation of the electrode–tissue interface location [12,13] and eventually hamper the recording ability of the electrodes. Our central nervous system (CNS) holds a central defence mechanism similar to our body’s natural mechanism. It initiates the microglia around the electrode–tissue interface site for recovering from the insertion injury [14,15]. Astrocytes activate within the first initial days for healing the injured wound [16]. In long-term implantation for chronic applications, micromotion occurs between the implanted electrodes and the neural tissues due to the mechanical mismatch induced by vascular pulsations [12]. A glial scar could be formed around the implantation sites, and over time, the electrical coupling or recording ability of the electrode could be reduced due to this formed scar. Due to these phenomena, the signal-to-noise ratio (SNR) and the number of neural tissues recorded in the interface decrease rapidly after the first several weeks post-implantation.

One essential consideration for implantable electrodes is a low Young’s modulus to reduce the glial scar or progression around the electrode, making any implantable devices mechanically similar to soft tissues [17,18]. Soft materials with low Young’s modulus can diminish the flexibility between the implantable electrodes and the neural tissue. Softer material can produce less mechanical strain on the surrounding tissue, resulting in a minimal inflammation response in chronic recording [19]. Anti-inflammatory drugs have been used as the coating on electrodes and have shown the potential to reduce glial encapsulation [20]. However, a flexible electrode also has drawbacks in several aspects. Firstly, a flexible electrode with anti-inflammatory drugs creates additional insertion difficulty due to its ultra-thin, flexible nature. Secondly, the glial scar can increase the electrode’s impedance and reduce the SNR, positively correlated with the noise [21,22]. In addition, the combination of high electrode impedance and the distributed capacitance between the electrode and the recording amplifier from the BCI device reduces the high-frequency response of the electrodes [23]. Lastly, the dimension of the electrode should be small enough on a specific brain region or neural location to minimize brain injury induced by the implantation. A large-sized electrode might damage many neurons during the insertion. Most previously developed implantable electrodes use conductive metals for clinical or neurobiology research, such as gold, platinum, iridium, stainless steel, and tungsten. Their surfaces are coated with a non-toxic, biocompatible insulating material on the electrode recording sites [10,24,25] to reduce inflammation, a common phenomenon in the chronic recording. 

Aizenberg et al. [26] have developed a slippery surface named slippery liquid-infused porous surface (SLIPS) through the inspiration of the *Nepenthis pitcher* plant by infusing the lubricant oil or slippery oil into a porous, rough, and lipophilic substrate. This oil-infused slippery surface demonstrates near frictionless properties, prolonged stability in liquid pressure situations, and shows liquid repellency against polar and non-polar liquids [27]. Moreover, it can recover structural integrity naturally through the natural oil mobility from the coating when the surface is damaged [28,29]. Therefore, it has been successfully studied for antifouling, highly “slippery”, and adhesion-free materials in various applications [30,31,32] and coating for medical devices [33,34]. However, such an ability has not yet been exhibited for ECoG electrodes without compromising the signal recording performance. 

Herein, we proposed a slippery-coated ECoG electrode with reduced adhesion for interfacing on the dura mater. The fabrication process and coating method were introduced, and the devices showed excellent electrochemical performance. Polyimide (PI) was used as a flexible substrate, and platinum (Pt) was used as the conductive material. The micro/nanofabrication method was used to fabricate a 10-channel ECoG electrode. Pt-gray was used for modifying the Pt surface to enhance the surface area of the electrode. Many other porous and rough coatings were applied on the microelectrodes, such as platinum black [35], Pt-gray [36], IrOx [37], carbon nanotubes (CNT) [38], graphene [39], etc. Pt-black can be used for the modified coating due to its porousness and lower density. Even so, it requires additional lead (Pb) to promote the electrodeposition plating, which is prohibited in medical or biomedical applications [40,41]. IrOx has poor adhesion on the Pt surface due to the lower stability. Nevertheless, Pt-gray has stronger mechanical strength and is suitable for medical applications [36]. A straightforward slippery coating method was developed on the electrode site. The SLIPS coating was hydrophobic, could repel artificial body fluids, and maintained stability for almost 12 weeks. The electrochemical characteristics showed that the proposed coated electrode reduced the impedance to 77%, resulting in a higher SNR than the bare electrode. The electrochemical fitting model and equivalent circuit parameters also proved the same result. The coating was biocompatible and toxic-free. In vivo study was conducted on a rat model and found that the SLIPS-coated ECoG electrode was excellent for recording the local field potential (LFP) and showed excellent performance while handling the noise signal. 

## 2. Materials and Methods

### 2.1. Fabrication Procedure of ECoG Electrode

**Figure 1:**
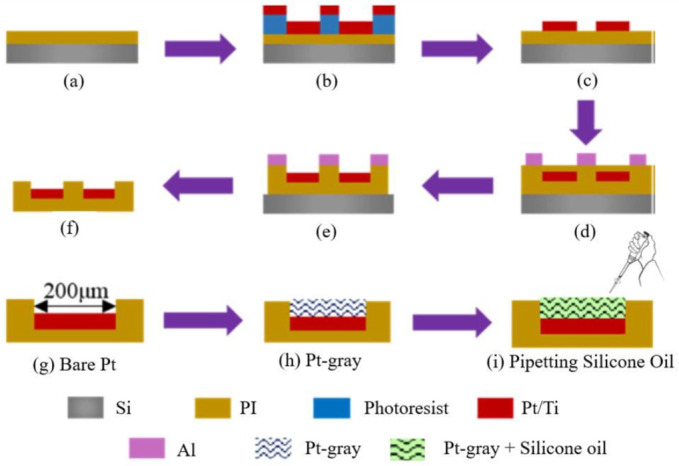
Step-by-step fabrication process of the ECoG electrode, where (**a**) PI deposition on Si wafer; (**b**) sputtering process; (**c**) lift off process; (**d**) passivation of PI layer; (**e**) RIE was used for exposing Pt; (**f**) electrode was released from Si wafer; (**g**,**h**) surface modification for the deposition of Pt-gray for creating the nano cone structures; and (**i**) Pipetting the silicone oil.

**Figure 2:**
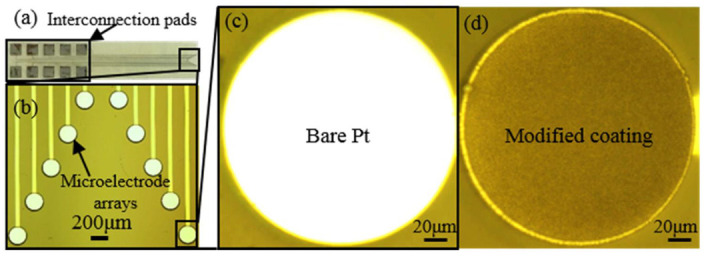
(**a**) Layout (2 × 5 arrays) of the fabricated ECoG electrode; (**b**) enlarged optical microscopic view of the recording sites (diameter was 200 µm). (**c**) Bare Pt surface before the surface modification and (**d**) modified surface with Pt-gray coating.

**Figure 3:**
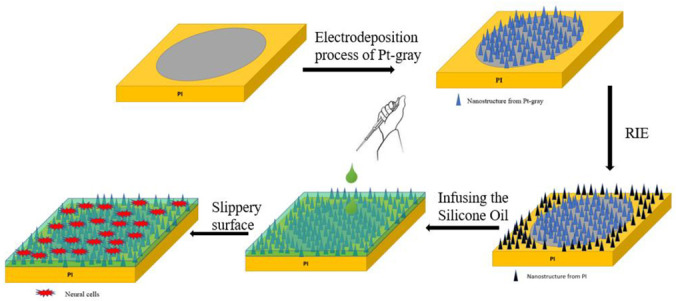
The schematic of the SLIPS coated procedure by pipetting the silicone oil on the electrode surface.

A thin Pt seed layer was deposited and patterned on the PI substrate to form an array of the electrode pad with 10 channels, where a single channel diameter was 200 µm. Briefly, spin coating was used to deposit the PI on the silicon (Si) wafer for curing with a thickness of 5 µm (Figure 1a). The sputtering and lift-off process was used to deposit the titanium (Ti) and Pt where the thickness was 20 nm and 100 nm, respectively (Figure 1b,c), with standard photolithography (EVG 610, Austria). After that, another PI passivation layer was developed using spin coating and cured. Finally, the reactive ion etching (RIE) was used for exposing the Pt (Figure 1d,e), and the ECoG electrode was released from the Si wafer (Figure 1f). After removing the electrodes from Si, optical microscopy was used to check the quality of the electrodes. Finally, O_2_ plasma reactive ion etching (RIE) was applied again for 10 min (flow rate = 20 sccm, pressure = 0~14 Pa, and power = 150 W) to create the nanocone structure on the PI substrate. Furthermore, O_2_ plasma reactive ion etching (RIE) is an excellent way to create the nanocone structure on the PI substrate (see Figure 2 and Figure 3). A layer-by-layer electrodeposition method (explained in Section 2.2) was used to deposit Pt-gray on the ECoG electrode to generate the nanocone, as shown in Figure 1h [36]. The fabrication process was easy to reproduce, had higher accuracy, was extremely scalable during manufacturing, and had a low unit cost during mass production. 

This nanocone enhanced the surface area and further reduced the impedance level. This Pt-gray-based nanocone helped create the porous surfaces, and the pipetting method was used for infusing a small amount (5 µL) of viscous silicone oil (Figure 1i) to create the slippery surface (explained in Section 2.2). The slippery surface or coating slides off readily in the aqueous solution or cell in contact. Silicone oil was purchased from Sigma-Aldrich China Inc. (CAS Number: 63148-62-9) and was used as received. It is famous for its superior stability, heat resistance, viscosity stability, thermal conductivity, chemical stability, high spreading power, and low surface tension. It can be mainly used in industrial products because of its distinctive properties, such as high lubricity, stable film formations, and non-toxicity. It is extensively used in lubricants, laboratories, electric insulators, anti-foaming, etc. Similarly, it is a slippery coating on the electrodes’ surface [42]. Figure 2 shows the optical images of the final ECoG electrode layout (2 × 5 arrays) and bare and modified electrode (Figure 2b,c) before and after the electrodeposition method.

### 2.2. Electrodeposition and SLIPS Coating Procedure on ECoG Electrode

A galvanostat (Gamry Reference 600) was used as an electrochemical workstation for all electrochemical experiments. A three-electrode electrochemical cell was used to deposit the Pt-gray composite on the electrode surface. The fabricated electrode was connected as a working electrode. A Pt sheet (1 cm × 1 cm) and Ag/AgCl electrode were used as counter and reference electrodes, respectively. Before starting the electrodeposition process, the electrode surface was cleaned carefully in acetone and 0.5M H_2_SO_4_ solution by 20 cycles of cyclic voltammetry (CV) (−0.2 V to +1.2 V vs. Ag/AgCl) with a scan rate of 100 mVs^−1^. The electrodeposited solution was prepared with a Pt chloride solution (7.5 mM PtCl_4_ (CAS Number: 13454-96-1), 10 mM (NH_4_)_2_PtCl_6_ (CAS Number: 16919-58-7), 25 mM NaH_2_PO_4_ (CAS No.: 7558-80-7), and 0.425 M Na_2_HPO_4_ (CAS Number: 7558-79-4) (purchased from Sigma-Aldrich, Shanghai, China)). The solution colour was amber, and pH was measured at ~7.9. Nitrogen gas was purged through the solution for degassing purposes. Finally, the chronoamperometry method was used to deposit the Pt-gray with a constant potential of −0.6 V vs. Ag/AgCl for 900 s. The Pt-gray created the nanocone structure on the electrode surface. After that, 5 µL silicone oil was applied by pipetting on the surface of the overall ECoG electrode to cover the whole probe, including the area of the marginal PI substrate and Pt electrode pads. The overall procedure is shown in Figure 3. The samples were kept on the plane surface for 2 h and held perpendicularly for 4 h to infuse the oil and remove the excessive oil later. 

Nepenthes pitcher plant has a microstructure to lock in an in-between liquid which acts as a repellent surface [43]. The created surface is in the aqueous phase and effective in dragging the insects from the rim to the digestive system by repelling the insects’ oiled feet [44]. Like the Nepenthes pitcher plant, SLIPS combines a well-matched nanocone structure with silicone oil to combine the liquid with microtextural roughness, forming a highly stable SLIPS coating which can be considered as a highly stable overlying coating.

### 2.3. Electrochemical Characterization

Electrochemical characterization was conducted using the same three-electrode systems using Electrochemical Impedance Spectroscopy (EIS) and cyclic voltammetry (CV). The EIS data were obtained with a 10 mV small Alternating Current (AC) signal, where the frequency was swept from 100 kHz to 1 Hz in phosphate-buffered saline (PBS) solution at room temperature. The CV data (−0.60 to +0.80 V vs. Ag/AgCl) were obtained with a scan rate of 50 mVs^−1.^ All the studies were performed on different categories of samples, which are named (i) before surface modification (S.M.) or bare electrode, (ii) after S. M. by Pt-gray, and (iii) SLIPS coated electrodes. ZView was used to find and fit the equivalent circuit model parameters. For each experiment, five samples were taken for experiments, and averaged data were analysed for final validation. 

### 2.4. Characterization

The surface modification of the ECoG electrode was analysed using a scanning electron microscope (SEM, SU-70, Hitachi, Tokyo, Japan). Contact angles (CA) and sliding angles (SA) were measured by dropping a liquid droplet (deionized water, PBS, Simulated Body Fluid (SBF)) on the surface of the sample. An optical contact angle meter (JC2000D, China) was used for these measurements. Three random locations were selected from the electrode surface to obtain the CA, and the mean value was calculated to determine the wettability. SA have taken similar experiments. Bare and SLIPS-coated electrodes were taken as samples from three different categories, and the means and standard deviation (SD) were calculated. To calculate each sample’s CA and SA, 4 μL DI water, PBS, and SBF were used as droplets. 

### 2.5. In Vitro Study (Cytotoxicity Study)

The cytotoxicity test conducts the biological evaluation and screening tests that use the tissue cells for in vitro study. The study observes the cell growth, reproduction, and morphological effects on the developed medical devices. The SLIPS-coated ECoG electrode is used as a target sample, with the bare electrode as the control group. Extract dilution methods were used with L929 cells described by many test reports, including ISO standards [45]. Next, 50 µL of Dulbecco’s Modified Eagle Medium (DMEM) (1.0 × 10^5^ cells/mL) was plated in each of the 24 wells and incubated under the standard culture conditions for the next 24 hours. In the extract the dilution test method, ISO standard procedures were used where 0.2 g of control and test sample as Perfluoro-n-octane (PFO) (C_8_F_18_) (CAS Number: 307-34-6) were added to 1 mL DMEM culture medium. Test samples were extracted from bare electrodes and SLIPS-coated electrodes. As the culture medium, 1 mL DMEM was used, and three replicates were used from each sample. Three batches (B-1, B-2, and B-3) were used for each sample from both the bare and SLIPS-coated electrodes. At 37 °C, the mixture was stirred for 24 h. After that, the culture medium L929 was removed by a slightly brisk inversion of the plates. Subsequently, each culture well-received 200 µL of the control and samples (bare electrodes and SLIPS-coated electrodes), as per the standard method. All the wells of the culture medium received 200 µL fresh DMEM. Finally, all the wells were incubated for 72 h in standard culture conditions and the viability of the L929 cell cultures of each well was measured using XTT (sodium 3′-(1-[phenylaminocarbonyl]- 3,4-tetrazolium)-bis (4-methoxy-6-nitro) benzene sulfonic acid hydrate) cytotoxicity assay. A Cell Proliferation Kit II (XTT) was purchased from Sigma-Aldrich, China and used following the manufacturer’s instruction. Then, 5 mL of a fresh mixture of XTT labelled reagent and 0.1 mL of the coupling reagents were mixed, and 50 µL of this mixture was mixed in each well. After incubation of 2 h, the absorbance study was completed, and the following equation was used to calculate the cell viability

(1) $$\mathrm{viability}=\frac{\mathrm{Test}\;\mathrm{Sample}}{\mathrm{Blank}\;\mathrm{Sample}}\times 100\%$$

A LUNA-II™ Automated Cell Counter was used for counting the cell to calculate the cell viability with Equation (1). 

### 2.6. In Vivo Recording (Acute Signal Recording) 

An in vivo experiment was conducted to verify the neural signal recording ability of the SLIPS-coated electrode. All the procedures involving animal use were approved by the institutional animal care and use committee (IACUC) of the Shenzhen Institute of Advanced Technology (SIAT) in Shenzhen, China and followed the ethical standards of the Animal Welfare and Use Guidelines of SIAT. 

**Figure 4:**
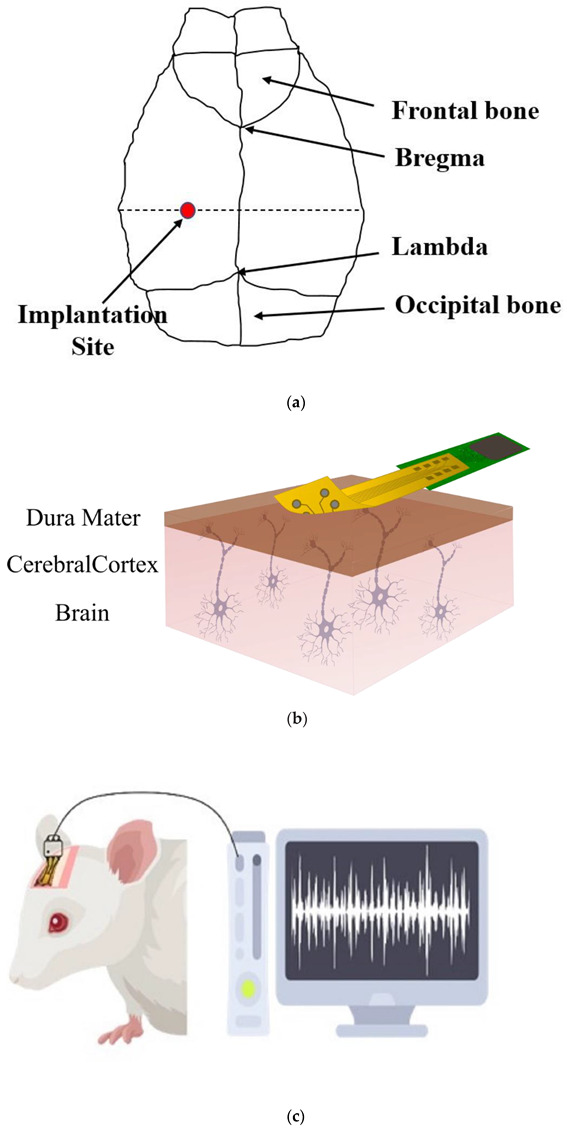
(**a**) Schematic diagram of the implantation site; (**b**) ECoG electrode implantation on the dura mater for neural recording; and (**c**) schematic of the experimental setup for ECoG signal recording.

Three twelve-week-old Sprague Dawley (SD) albino male rats were used for this experiment. The rats were anaesthetized using 4% isoflurane (CAS Number: 26675-46-7) for induction and 2% isoflurane during the surgery, which was applied by an isoflurane vaporizer (R540 Mice and Rat Animal Anesthesia Machine, RWD, China). The anaesthetized rats were placed on a rail-mounted Rat Stereotaxic Instrument (RWD, China) for surgery, and the hair was removed from the skull. Three bare electrodes and three SLIPS-coated electrodes were used for implantation purposes to record the acute signal from the motor cortex area of the left lobe. The recording sites were considered the epidural area of the brain (as illustrated in Figure 4a). The target site was found in the atlas of Paxinos and Franklin [46].

Additional drilling was performed to provide the ground screw (frontal bone) (from Figure 4a), including the target site, which was drilled for ECoG electrode implantation. The developed electrode is an epidural ECoG electrode. Therefore, the electrode was interfaced on the outer surface of the dura mater (Figure 4b). The bare and ECoG electrodes were implanted simultaneously in the same rat for accurate recorded neural signals comparison. The neural signals were recorded by the commercial recording system (Intan RHD recording head stage (32 Channels [47] and RHD USB interface board [48])) (Figure 4c). The rats were anaesthetized during the experiment to restrict their movements, free from electrical noises caused by their natural behaviour. A customized printed circuit board (PCB) interface unit was also developed for connecting the ECoG electrode with the head stage. After that, the signals were analysed by the OriginLab signal processing tool and MATLAB signal analysing tool for processing the signals and identifying the ECoG signals from the recorded signals. 

## 3. Results and Discussions

### 3.1. Electrodeposition Process and Morphology Study

**Figure 5:**
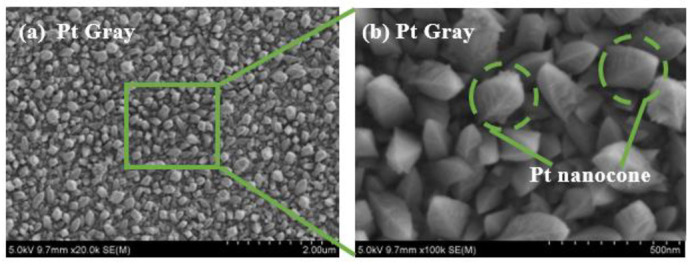
SEM of Pt-gray and porous surface (**a**,**b**) created for nanocone structure.

After finishing the fabrication steps, it is crucial to have good adhesion on the Pt layer on the electrode’s recording sites. Nano-shaped Pt-gray has been created, providing a rough surface and excellent adhesion on the substrate. It is essential to control the voltage, time, and molar ratio of the Pt chloride solutions, which are critical factors, to obtain optimal morphology, the impedance of the electrode, stability of the nanostructures, and excellent performance of the nanostructured Pt-gray. The porous Pt-gray has two significant advantages: creating a large surface area that eventually reduces the overall impedance [49] and creating the nanocone structure where the silicone oil can be infused [26]. The optimized voltage was found as −0.6 V vs. Ag/AgCl to obtain the nanocone structure, which can be seen in Figure 5. The molar ratio of NH_4_^+^ to Pt_4_^+^ controls the nanocone-shaped microstructure where NH_4_^+^ and Cl^−^ were used as crystal modifiers [50]. Without the NH_4_^+^, it would be challenging to create the nanocone structure due to a higher rate of forming crystal structure. Using the Pt chloride solution from (NH_4_)_2_PtCl_6_ (10 mM) and PtCl_4_ (7.5 mM) also helped to develop a stable microstructure for more extended stability of the final coating. 

The microstructure of the surface area can primarily influence the electrochemical behaviour of the coating on the electrode. The microstructure’s surface area significantly impacts reducing the impedance, which is essential for recording the neural signal (Figure 5a). From Figure 5, the morphology clearly shows the microstructures of Pt-gray deposition by constant potentials. The rough surface was formed by nanocone structures which are also essential to infuse the silicone oil for creating the SLIPS coating. 

### 3.2. Evaluation of Electrochemical Performance

**Figure 6:**
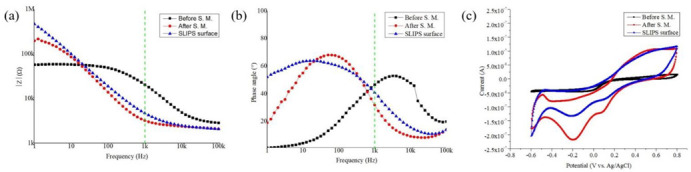
Electrochemical performance of the proposed electrode before the surface modification, after the surface modification by Pt-gray, and SLIPS coating. (**a**) Electrochemical impedance profile, (**b**) phase angle profile, and (**c**) CV analysis at a sweep rate of 50 mV/s.

It is crucial to improve the electrochemical surface area of the ECoG electrode by reducing its impedance, which eventually reduces the impedance of the electrode–electrolyte neural interface during the neural signal recording. Reducing the impedance can increase the SNR and decrease the thermal noise. Therefore, it is essential to have the impedance value of the electrode be less than a few hundred kΩ for seamless neural signal recording [51]. Figure 6a,b shows the electrochemical impedance and phase change. At 1 kHz, the bare electrode (named before the surface modification (S.M.)) impedance of 20.38 kΩ. Pt-gray and the nanostructure modified the electrodes’ surface, which enhanced the overall surface area and changed the impedance to 3.21 kΩ (reduction of 84.24%). A typical SLIPS-coated electrode impedance is slightly increased compared to the electrode with Pt-gray only. The impedance increment is due to the additional silicone oil layer over the nanocone structure during the formation of a SLIPS coating. This additional layer or SLIPS coating works as an insulator, degrading the electrodes’ performance. With the SLIPS coating, the impedance increased from 3.21 kΩ towards 4.68 kΩ. Yet, the impedance reduction is almost 77% compared to the bare electrode, which remains excellent for chronic neural recording applications. The results are summarized in Table 1.

The redox characteristics curve for the ECoG electrode was studied further using cyclic voltammetry (CV), as shown in Figure 6c. The charge storage capacity (CSC) of any microelectrode or implantable electrode is a crucial indicator to justify the performance of neural stimulation. It is estimated by using the time integral of the cathodic current. The CSC was calculated using the following formula [52]:

(2) $$\mathrm{CSC}=\frac{1}{\mathrm{vS}}\int_{\mathrm{Ec}}^{\mathrm{Ea}}\mathrm{IdE}\;$$

where E is considered the potential (V vs. Ag/AgCl), I is the measured current (A), E_c_ and E_a_ are the cathodic and anodic potential limit, and S is the geometric area of the electrode (cm^2^). The diameter of the surface area of the electrode was 200 µm, and, therefore, S is calculated as 3.14159 × 10^−4^ cm^2^. Finally, v is considered from the scan rate. The calculated CSC is tabulated in Table 1. It is found that the CSC increases 5.2 times compared to the counterpart without any surface modification. It is due to the increment in the effective surface area by the electrodeposition process of Pt-gray. The SLIPS-coated electrode has also shown a similar CSC performance. This expected result proved that the developed electrode would be excellent for neural stimulation. However, this paper focuses only on neural recording, and all the characterizations are designed to validate the recording results.

**Figure 7:**
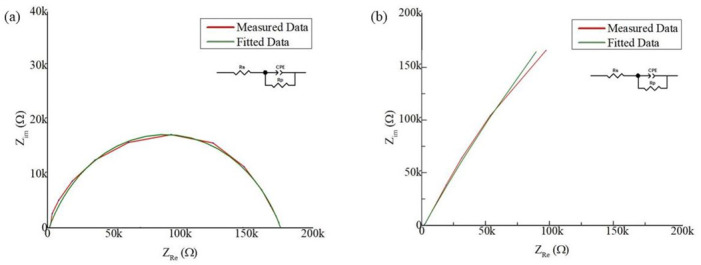
Equivalent circuit of the electrode–electrolyte for (**a**) bare electrode or before the surface modification of the electrode and (**b**) the SLIPS-coated electrode.

The equivalent circuit parameters from the EIS model and fitting curve for the bare electrode and SLIPS-coated ECoG electrode are shown in Figure 7. The fitting parameters from the simulated curve are tabulated in Table 2. R_s_ and R_p_ are considered solution resistance and charge transfer resistance, respectively. CPE is a constant phase element introduced to present an electrical double-layer capacitance (EDL). The EDL is relatable to the non-homogeneity of the electrode surface and nano distribution at the liquid–solid interface [37,53]. CPE was used instead of the ideal capacitor due to the non-ideal capacitance response. The impedance from CPE can be given by $Z_{CPE}=\frac{1}{A{\left(i\omega \right)}^{n}}$; CPE is composed of constant *A* and dispersive *n*; *ω* is the angular frequency (rad/s). *A* and *n* are frequency-independent parameters, where 0 ≤ n ≥ 1. CPE would be considered as an ideal capacitor if n = 1 or purely resistive if n = 0 [54,55]. From Table 2, it is shown that *n* is increased from 0.75 to 0.86 of the increased roughness of Pt-gray deposition. The charge transfer resistance is also decreased from 5.73 kΩ to 2.80 kΩ, which proves the surface modification’s roughness. The solution resistance is stable for all the cases, around 2.1 kΩ. In conclusion, it can be said that surface modification has significantly improved the electrode’s performance and would be excellent for neural recording. The fitting models’ errors are less than 5%, confirming that the equivalent circuit models are modelled as expected. 

### 3.3. Characterization of SLIPS Coating

**Figure 8:**
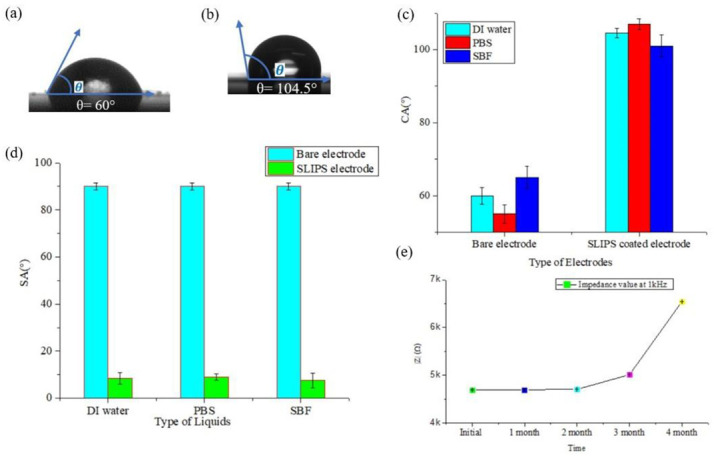
DI water droplet on the SLIPS surface (**a**) before the SLIPS coating, which is hydrophilic, and (**b**) after the SLIPS coating, which is hydrophobic. (**c**,**d**) CA and SA for various fluids where the samples are taken from three different locations. (**e**) The long term stability test for four months.

Hydrophobicity is an essential parameter in understanding the wettability of any surface. The Pt-gray nanocones have enhanced the effective surface area and reduced the impedance. Meanwhile, SLIPS coating has decreased the effective surface area and reduced the wettability. In Figure 3, the O_2_ plasma treatment has also changed the wettability of the marginal area of the PI substrate. It is necessary to create roughness on the substrate site, which eventually reduces the overall wettability of the developed ECoG electrode. In Figure 8a,b, it is seen that the hydrophobicity has increased significantly due to the SLIPS coating. Initially, the CA was 60°, which is considered a hydrophilic surface. After the surface modification by Pt-gray and SLIPS coating, the wettability has reduced to 74.2%, and the surface became hydrophobic due to the higher CA (≥90°). The other liquids have shown similar results where the CA did not change much compared to the bare electrodes. The SA test has also shown that the surface became hydrophobic and liquids have slid off from the surface rapidly. These results are suggestive of the nonadhesive properties of SLIPS coating. A monthly measurement of the impedance also tested the stability after four months. In Figure 8e, it is seen that the impedance level was stable for the first three months; after that, the impedance level increased. The coating is stable for three months and can meet most chronic applications’ requirements. The nanocone structure might start to deteriorate after three months, so the impedance is seen to be increased. The SLIPS-coated ECoG electrode has demonstrated the prolonged and lifetime stability of impedance level compared to the bare electrode, which is close to frictionless, similar to exploring the biomimetic approach of the frictionless Nepenthes pitcher plant’s surface. The SLIPS-coated electrode also showed long-term stability by reducing the adhesion of various liquids, including biological substances, between the lubricant and the electrode’s surface.

### 3.4. Evaluation of In Vitro Study (Cytotoxicity Study)

**Figure 9:**
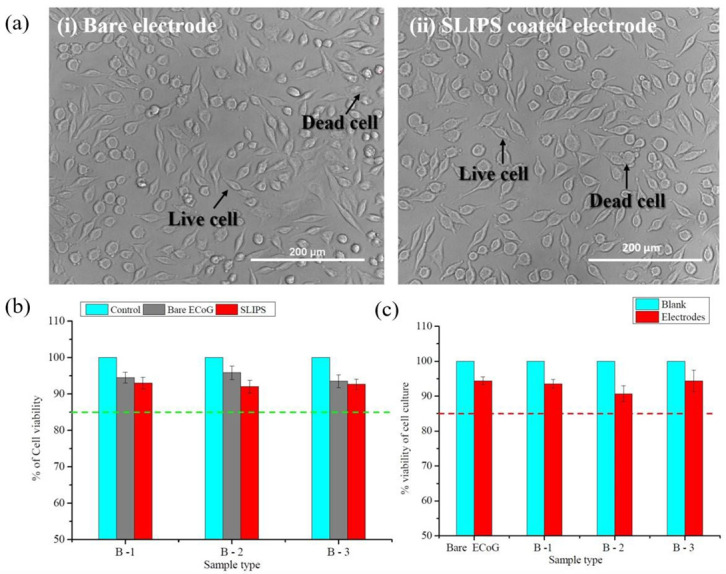
(**a**) Optical microscopy image of cell viability test where live and dead cells are marked by black indicators. (**b**) Cell viability test by cell counting for control, bare, and SLIPS coated electrode, and (**c**) absorbance study of blank, bare ECoG electrode, and various batches (B-1, B-2, and B-3) of SLIPS coated electrodes.

The optical images in Figure 9a show the healthy (live cells) and unhealthy cells (dead cells) due to the toxicity effect of the SLIPS coating. It is seen that damaged cells are relatively few compared to healthy cells. The bare and SLIPS-coated electrodes showed similar results in Figure 9b,c. Figure 9b is generated from the cell counting results, and Figure 9c is generated from the absorbance study. Both figures show that more than 85% of cells survive for the blank and bare electrodes (mean ± SD). A two sample t-test was performed between the different batches (B-1, B-2, and B-3) of electrodes (bare ECoG electrode and SLIPS-coated ECoG electrodes). From the analysis for Figure 9c, *p* = 0.37, which is greater than the significance level, α = 0.05. It means there is no significant difference in the cell viability for both the bare and SLIPS-coated ECoG electrodes. Similar results for Figure 9c are found from a two-sample t-test. No additional toxicity effects occurred due to the developed coating on the electrode’s surface. All the samples have shown similar results, indicating that the toxicity effect is slight and acceptable. The results can be considered good, showing that the coating has no cytotoxic potentiality and could be biocompatible with the human body. 

### 3.5. Evaluation of In Vivo Recording (Acute Signal Recording) 

**Figure 10:**
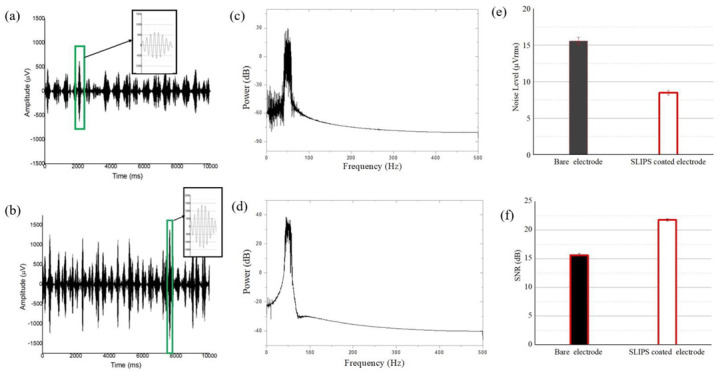
In vivo recording of the LFP signal from the (**a**) bare electrode, (**b**) SLIPS-coated electrode, (**c**) power density of bare ECoG electrode, (**d**) power density of SLIPS-coated ECoG electrode, (**e**) noise voltage level, and (**f**) SNR from both electrodes.

Figure 10 shows the in vivo signal recording from the bare ECoG electrode and SLIPS-coated ECoG electrode. From Figure 10a,b, it is seen that the noise level is reduced significantly as a result of the surface modification. Figure 10c,d also showed the power density of the recorded LFP from the bare and SLIPS-coated ECoG electrodes, respectively. Due to the impedance difference, the bare electrode recorded more noised signals than the coated electrode. The signal-to-noise ratio (SNR) is also improved and sufficient to record the ECoG signal. The noise level was recorded by dipping the electrode (bare and SLIPS coated) in the PBS solution and recording the signal for 1s. It was found that the noise level for the bare electrode was an average voltage (BE_Vrms), 15.55 µV, and was reduced to (SE_Vrms) = 8.5 µV for the SLIPS coated electrode (Figure 10e). The decrease induces this noise level reduction in the overall impedance level from 20.38 kΩ to 4.68 kΩ (see Table 1). The LFP signal was recorded from the cortical surface area of the rat. It is considered that the average peak-to-peak amplitude of the recorded neural signal is estimated at around 800 µV for the bare electrode and 1800 µV for the SLIPS-coated electrode (see figure inset in Figure 10a,b). The SNR value calculated from the LFP signal increased from 15.60 dB to 21.76 dB (Figure 10f). The results indicated that the SLIPS-coated ECoG electrode could reduce noise due to the proposed coating methods, improve the ECoG electrode’s electrochemical properties, and improve the recording quality of neural signals. 

The ECoG signal amplitude is often recorded in the mV range [56]. Therefore, the effect of the thermal noise is significant during the recording tasks, which is related to the double-layer capacitance between electrode–electrolyte and electronic noise [57]. The thermal noise can be calculated by using the, $V_{rms}=\sqrt{4\mathrm{kTZB}}$, where k is the Boltzmann constant, T is the absolute temperature, Z is real impedance from the electrode at 1 kHz, and B is the noise band width or operating frequency. In Table 2, Rp is placed to calculate the ratio of the noise voltage Vrms, where the ratio is 1.43. This is similar to the experimental noise level ratio, BE_Vrms/SE_ Vrms = 1.82. Since both the trends are similar, it can be concluded that noise level of the SLIPS coated electrode is significantly reduced and verified by EIS analysis. 

Overall, the developed ECoG electrode with the SLIPS coating showed an excellent impedance profile compared to other similar electrodes (5–10 kΩ [58], 40–160 kΩ [59], and 1–5 kΩ [60]). It also showed reduced noise level, stable coating for more than three months, biocompatibility, and potential for future use for chronic neural recording applications. 

## 4. Conclusions

The fabrication of the 10-channel PI/Pt-based ECoG electrode is proposed. The electrode surface is modified with Pt-gray to create the nanocone structure, reduce impedance, and develop a Nepenthes pitcher plant-like slippery structure on the recording surface. The surface modification has reduced the impedance to 77% and improved the SNR from 15.60 dB to 21.76 dB. The procedure of the slippery coating on the electrode surface is easy to fabricate. The coating showed excellent hydrophobicity, stability, and adhesion with various fluids. The cytotoxicity study also showed that the coating is biocompatible and not harmful to cells. The in vivo recording has shown excellent neural signal recording performance, and the noise level calculation matches the equivalent circuit parameters. Overall, the proposed SLIPS-coated epidural ECoG electrode can provide a neural interface on the dura mater and has the potential for chronic neural recording applications. The validation of chronic recording will be tested on an animal model in future works and reported in our future article. 

## Figures and Tables

**Table 1:** Comparison of reduction in impedance at 1 kHz and CSC of the proposed ECoG electrode for various sample conditions.

Sample Condition	Initial Impedance (at 1 kHz)	Reduction of Impedance (%)	CSC/mC cm^2^
Before S. M.	20.38 kΩ	--	2.07
After S. M.	3.21 kΩ	84.24	10.77
SLIPS coating	4.68 kΩ	77.03	10.8

**Table 2:** Fitting parameters from the EIS model.

Type of Electrode	R_p_ (Charge Transfer Resistance), kΩ	A(F)	n	Error (R^2^) (%)
Before the surface modification	5.73	5.18 × 10^−8^	0.75	3.18
SLIPS coating	2.80	5.01 × 10^−7^	0.86	1.93

## Data Availability

Not applicable.
